# Detection and Quantitative Assessment of Arthroscopically Proven Long Biceps Tendon Pathologies Using T2 Mapping

**DOI:** 10.3390/tomography9050126

**Published:** 2023-08-23

**Authors:** Patrick Stein, Felix Wuennemann, Thomas Schneider, Felix Zeifang, Iris Burkholder, Marc-André Weber, Hans-Ulrich Kauczor, Christoph Rehnitz

**Affiliations:** 1 Diagnostic and Interventional Radiology, University Hospital Heidelberg, Im Neuenheimer Feld 420, 69120 Heidelberg, Germany; 2 Institute of Diagnostic and Interventional Radiology & Neuroradiology, Helios Dr. Horst Schmidt Clinics Wiesbaden, Ludwig-Erhard-Straße 100, 65199 Wiesbaden, Germany; 3 Center for Orthopedics, Trauma Surgery and Spinal Cord Injury, University Hospital Heidelberg, Schlierbacher Landstraße 200A, 69118 Heidelberg, Germany; 4 Ethianum Clinic Heidelberg, Voßstraße 6, 69115 Heidelberg, Germany; 5 Department of Nursing and Health, University of Applied Sciences of the Saarland, 66117 Saarbruecken, Germany; 6 Institute of Diagnostic and Interventional Radiology, Pediatric Radiology and Neuroradiology, University Medical Center Rostock, Ernst-Heydemann-Straße 6, 18057 Rostock, Germany

**Keywords:** T2 mapping, long biceps tendon, tendinopathy, arthroscopy

## Abstract

This study evaluates how far T2 mapping can identify arthroscopically confirmed pathologies in the long biceps tendon (LBT) and quantify the T2 values in healthy and pathological tendon substance. This study comprised eighteen patients experiencing serious shoulder discomfort, all of whom underwent magnetic resonance imaging, including T2 mapping sequences, followed by shoulder joint arthroscopy. Regions of interest were meticulously positioned on their respective T2 maps, capturing the sulcal portion of the LBT and allowing for the quantification of the average T2 values. Subsequent analyses included the calculation of diagnostic cut-off values, sensitivities, and specificities for the detection of tendon pathologies, and the calculation of inter-reader correlation coefficients (ICCs) involving two independent radiologists. The average T2 value for healthy subjects was measured at 23.3 ± 4.6 ms, while patients with tendinopathy displayed a markedly higher value, at 47.9 ± 7.8 ms. Of note, the maximum T2 value identified in healthy tendons (29.6 ms) proved to be lower than the minimal value measured in pathological tendons (33.8 ms), resulting in a sensitivity and specificity of 100% (95% confidence interval 63.1–100) across all cut-off values ranging from 29.6 to 33.8 ms. The ICCs were found to range from 0.93 to 0.99. In conclusion, T2 mapping is able to assess and quantify healthy LBTs and can distinguish them from tendon pathology. T2 mapping may provide information on the (ultra-)structural integrity of tendinous tissue, facilitating early diagnosis, prompt therapeutic intervention, and quantitative monitoring after conservative or surgical treatments of LBT.

## 1. Introduction

Shoulder pain is a common clinical issue and a known cause of disability, which can lead to significant functional impairment and thereby compromise the overall quality of patients’ lives [1,2]. Pathologies of the long biceps head tendon (LBT) are a significant source of shoulder pain, not only because of their association with other shoulder injuries, but also because of their tendency to become chronic [3,4]. In addition to middle-aged or older adults, overhead athletes are also known to be at risk for developing LBT tendinopathy, making them particularly susceptible to chronic shoulder pain given their usually young age [5,6].

In addition to clinical examination, imaging techniques, especially non-contrast magnetic resonance imaging (MRI), are central to the evaluation of the shoulder joint. While a variety of common shoulder pathologies can be detected via MRI with relatively high diagnostic accuracy, several studies have reported suboptimal diagnostic performance regarding pathologies of the LBT, which are usually visualized in more advanced stages of tendinopathy or rupture [7,8,9,10]. In an arthroscopy-controlled study, Dubrow et al. reported a sensitivity of only 56.3% for non-contrast MRI in detecting complete LBT tears [11]. The diagnostic performance in detecting partial tears was even lower, with a sensitivity of 27.7%. As reported by Tadros et al. and De Maeseneer et al., even the additional performance of MR arthrography did not show improvement in the detection of LBT pathologies, with sensitivities ranging from 15 to 38% [12,13]. In addition, the visualization of LBT may be difficult in conventional 2D MRI sequences due to the arcuate course of the tendon through the glenoid joint in conjunction with its typically small size when using slice thicknesses of 3 mm, making it susceptible to imaging artifacts such as the partial volume effect. Early LBT pathologies can only be detected to a very limited extent by means of common morphological MRI sequences, and a diagnostic tool for early diagnosis is lacking, which appear to be even more problematic, as tendon degeneration seems to be a chronically progressive process [14,15].

Functional MRI sequences, such as T2 or T2* mapping, have mainly been used for the evaluation of ultrastructural changes affecting the collagen network of articular cartilage and have been shown to detect and quantify early degenerative changes in various joints [16,17,18]. Because changes in proteoglycans, and thus, water content are known histologic signs of early osteoarthritis (OA), quantitative MRI sequences with measurements of the T2 relaxation time have been investigated in previous studies of OA and its progression [19,20]. In view of the promising results, these sequences were also included in a multicenter longitudinal study called the Osteoarthritis Initiative, which found an association between elevated T2 values and knee pain along with early signs of OA [21]. In addition to joint degeneration, Kasar et al. reported increased T2 values in the sacroiliac joints of patients with active and even inactive axial spondyloarthropathies, making T2 mapping sequences potentially useful for detecting inflammation-related tissue changes [22]. Since microscopic mucoid degeneration and disruption of the 3D collagen network in tendinopathic LBT specimens have been reported as histopathological correlates of hyperintense signal changes in T2-weighted sequences, T2 mapping might also be a viable tool for assessing and especially quantifying damages to tendinous structures [23]. To our knowledge, T2 mapping has rarely been systematically studied for the assessment of LBTs or tendons in general, and the few existing studies have not yet validated this technique using arthroscopy [24,25].

Thus, the purpose of this study was to investigate the ability of T2 mapping to detect arthroscopically proven pathologies of the long biceps tendon and to quantify the correlating T2 values for damaged and healthy tendons.

## 2. Materials and Methods

### 2.1. Participants and Inclusion

Over a period of three months, this study enrolled 19 consecutive patients with shoulder pain who were referred to our radiology department for preoperative MRI assessments between September and November 2017. MRI and shoulder arthroscopy were indicated when the patient complained of significant shoulder pain, severe enough to interfere with activities of daily living, and for which conservative therapy, including physical therapy and analgesic medications, did not improve the overall pain level. In patients with negative MRI examination, the indication for exploratory shoulder arthroscopy was based on the combination of symptom severity, clinical examination, and ineffective conservative therapy. Patients with endoprosthetic joint replacement or osteosynthetic material at the proximal humerus, advanced osteoarthritis (OA; Kellgren–Lawrence score >1), a previous shoulder joint surgery, or an age of less than 18 years were excluded from the study. In addition, one patient was excluded who mistakenly underwent a routine MRI protocol without T2 mapping sequences, leaving 18 patients for inclusion in the study. The study was approved by the Institutional Review Board of Heidelberg University (S-081/2010) and was conducted in accordance with the Declaration of Helsinki. Written informed consent was obtained from all patients after they were informed of the nature of the examination.

### 2.2. Study Design

After enrollment, each participant underwent the index test, which included a T2 mapping MRI sequence at 3 Tesla. Subsequently, the reference examination, a shoulder arthroscopy, was performed within a maximum period of 6 days (mean: 4 days). Based on the arthroscopy findings, the study population was divided into two subgroups: “healthy subjects” and “patients with confirmed LBT pathology”. With respect to item 5 from the latest version of the STARD guidelines published by the EQUATOR network (Centre for Statistics in Medicine (CSM), NDORMS, University of Oxford, Oxford, UK) in 2015, this study can, therefore, be defined as a prospective observational study [26].

### 2.3. MRI Protocol and T2 Mapping

Each participant underwent imaging with a 3-Tesla MRI system with a 70 cm wide gantry and an 18-channel imaging matrix (Magnetom Verio, Siemens Healthineers, Erlangen, Germany). The examination was conducted in the supine position with the shoulder joints kept stabilized in external rotation to ensure that the joint was positioned as isocentrically as possible. A standard house-internal MRI protocol was used to morphologically assess the joint structures, including the proton density-weighted fat-saturated sequences as well as the T1- and T2-weighted sequences without fat saturation. For the acquisition of the oblique coronal views, the sequences were acquired perpendicular to the glenoid fossa, whereas the oblique sagittal sequences were aligned parallel to the glenoid articular surface. A manufacturer-supplied oblique coronal multiecho spin-echo T2 mapping sequence (syngo MapIT, Siemens Healthineers, Erlangen, Germany) was used as the primary sequence of interest. In this sequence, a pixel-by-pixel, monoexponential, non-negative least squares analysis was conducted to determine the T2 relaxation times from the T2 parameters. This analysis resulted in a color-coded T2 map, which could then be used for subsequent analysis.

The MRI protocol used in this study represents our in-house standard protocol augmented by the T2 mapping study sequence, and is based on a previous study by our research group investigating the diagnostic performance of T2 mapping with respect to the detection of superior labral anterior to posterior (SLAP) lesions [27]. For more details on the MRI study protocol and the T2 mapping sequence, see Table 1.

### 2.4. Image Analysis and Definition of LBT Pathologies

A picture archiving and communication system (Centricity PACS, v. 4.0; GE Healthcare IT Solutions, Barrington, IL, USA) was used to evaluate the morphologic sequences by two independent radiologists with 19 (CR) and 6 (FW) years of experience in musculoskeletal MRI. Both gained experience in the MRI evaluation of the shoulder joint through several training courses as well as by participation in previous studies with functional and quantitative MRI imaging techniques for (fibro)cartilaginous structures. The parameters for slice selection, magnification, and windowing were set by both radiologists, and the ambient light was minimized during the reading sessions. To ensure objectivity, the patients’ names, clinical data, arthroscopy results, and ratings from the other reader were blinded for both radiologists. As all study participants were informed about the additional study sequence and their upcoming shoulder arthroscopy, they were not blinded.

Proton density-weighted, fat-saturated sequences were utilized to classify the LBT as either normal or damaged. Focal or longitudinal changes in signal intensity, a significantly increased tendon diameter, marked fraying or contour irregularities, and partial or complete tears of tendon substance were defined as LBT tendinopathy. If none of these diagnostic features were present, the LBT was considered normal.

### 2.5. Placement of Regions of Interest

**Figure 1:**
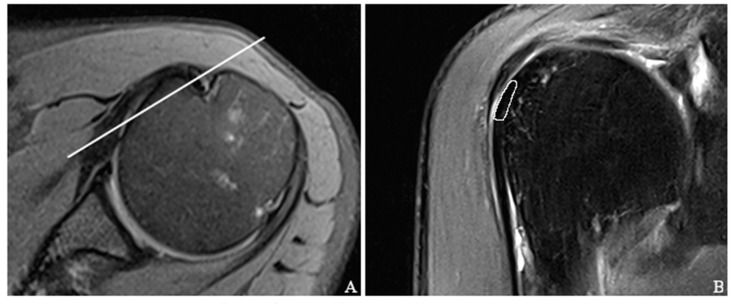
Axial (**A**) and oblique coronal (**B**) proton-density-weighted sequences showing the slice positioning (white line in (**A**)) used to place the region of interest (ROI). As outlined by the white circle in (**B**), the sulcal portion of the long biceps tendon was chosen as the primary region of interest and subsequently transferred onto a color-coded T2 map.

First, an oblique coronally orientated proton-density-weighted fat-saturated sequence dissecting the bicipital groove was used to identify the LBT and decide upon the optimal position for the placement of the region of interest (ROI). The ROI was placed in the section in which the largest longitudinal diameter of the sulcal part of the LBT was depicted and no partial volume effect was present (Figure 1). To further reduce the possible effects of artifacts or imperfect ROI placement, each measurement was performed three times, and the average T2 relaxation times were used for analysis. In order to obtain precise measurements, the regions of interest (ROIs) were carefully placed on the first echo images acquired using a multi-echo-spin-echo T2-weighted sequence. Subsequently, the ROI was duplicated onto a color-coded parametric T2 map after visual comparison with the proton-density-weighted fat-saturated sequences and further adjusted, if necessary, to exclude artifacts or non-tendinous structures, such as the humeral cortex or deltoid muscle.

### 2.6. Arthroscopy

Two experienced orthopedic surgeons performed shoulder arthroscopy on all patients. The operations were performed under general anesthesia with the patients seated at an angle of 30–90° above the horizontal plane and the head fixed in a headrest. During the arthroscopy, both the labroligamentous and cartilaginous joint structures were assessed. A standardized questionnaire was used to evaluate the intra-articular portion of the LBT, including its extension into the sulcal portion. If medically indicated, a ventral approach was made for surgical intervention. The diagnosis of LBT tendinopathy was defined by a marked fraying of the tendon substance or significant changes in the tendon diameter. Partial tears were defined as a disruption of a single or multiple tendon fibers, whereas complete tears were defined as a disruption of the entire thickness of the LBT. In terms of the statistical calculations, partial and complete tears were also considered tendinopathic changes.

### 2.7. Statistical Analysis

The analysis of the patient’s demographic data was carried out in a descriptive manner, while the analysis of the continuous variables was summarized as the mean values, median, and associated extrema. The frequency and percentage were calculated for the qualitative variables. For comparison of the patients’ ages between the groups with and without LBT tendinopathy, the Wilcoxon two-sample test was used.

For analysis of the T2 mapping values of the sulcal part of the LBT, three measurements were performed by the two readers and repeated within a time interval of 7 days for calculation of the intra-rater agreement. Statistical analysis of the T2 imaging was carried out descriptively using summary statistics and interpreted exploratively. A comparison of the groups with and without lesions was performed using a two-sample *t*-test. The *p*-values were presented as two-sided *p*-values, and the level of significance was set to 5%. To assess whether the T2 mapping values were normally distributed, the Shapiro–Wilk test was used.

We performed an analysis of the diagnostic performance of T2 mapping for tendinopathic changes in the LBT or LBT lesions and reported the estimates and exact 95% confidence intervals for the sensitivity, specificity, positive predictive values, and negative predictive values. As complete separation of the data occurred, no ROC curves were plotted for the accuracy assessment.

As the raters were viewed as a random selection of observers drawn from a larger pool of possible observers, the intraclass correlation coefficient (ICC) was utilized to assess both the inter- and intra-reader agreement. In order to gauge interrater reliability, we employed a two-way random-effect model considering the subject and rater as random effects to estimate the ICC and 95% confidence intervals, following the methodology outlined by Shrout and Fleiss [28]. Data analysis was carried out using SAS for Windows (version 9.4; SAS Institute Inc., Cary, NC, USA) and R version 3.5.1 (www.cran.r-project.org, accessed on 10 May 2022) by a qualified statistician who was independent of the study.

## 3. Results

### 3.1. Demographics and Arthroscopic Evaluation of the Long Biceps Tendon

A total of 12 (66.7%) out of the 18 patients included were male, and 6 (33.3%) were female. The mean patient age was 52.4 ± 14.72 (range, 22.0–67.0) years. On average, patients with LBT tendinopathy were slightly younger than the healthy subjects (46.3 ± 17.2 (range: 22–64) years vs 60.0 ± 5.0 (range: 52–67) years). Out of the eight patients with arthroscopically proven LBT tendinopathy, seven (87.5%) were male and only one (12.5%) was female. In Table 2, an overview of the patients’ demographic characteristics is presented, categorized by whether LBT tendinopathy was present or not.

A total of 10 persons (55.6%) had healthy long biceps tendons, whereas 8 patients (44.4%) showed tendinopathic changes. Out of the eight patients with pathologic long biceps tendons, the majority showed minor lesions or partial tears (*n* = 7; 87.5%), whereas one patient (12.5%) was diagnosed with a complete tear. Using the conventional morphological MRI sequence, all eight patients with arthroscopically proven tendinopathy were detected by both readers.

### 3.2. T2 Mapping of the Long Biceps Tendon

**Figure 2:**
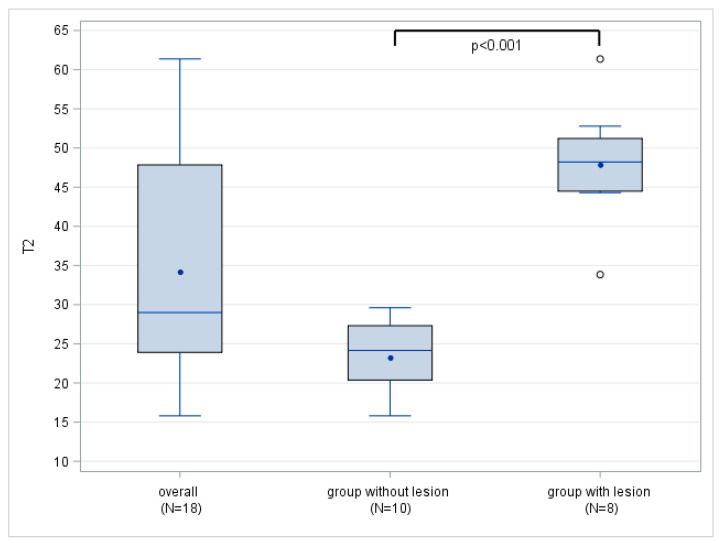
Boxplots of overall T2 mapping values (ms) in healthy labrum and patients with LBT lesion. Note the complete separation of T2 values between patients with LBT lesions and healthy subjects.

In matching the arthroscopy results, eight LBT lesions were detected in the MRI, and no additional lesions were found intraoperatively. The mean T2 values show a significant difference between the patients with LBT tendinopathy, with 47.9 ± 7.8 ms, and the healthy subjects, with 23.3 ± 4.6 ms (*p* < 0.001). In the subgroup of healthy individuals, the maximum T2 value measured was 29.6 ms, which was found to be lower than the minimum T2 value recorded in patients with confirmed LBT tendinopathy (33.8 ms). This difference led to a complete separation of the T2 mapping values between the normal and pathological tendon substance, as depicted in Figure 2. As a result, all cut-off values between 29.6 ms and 33.8 ms showed sensitivities and positive predictive values of 100%, with 95% confidence intervals (CIs) (63.1–100.0%), along with specificities and negative predictive values of 100%, with 95% CIs (69.2–100.0%). For a more comprehensive view, Table 3 illustrates the mean T2 mapping parameters for patients with LBT tendinopathy and for healthy individuals.

**Figure 3:**
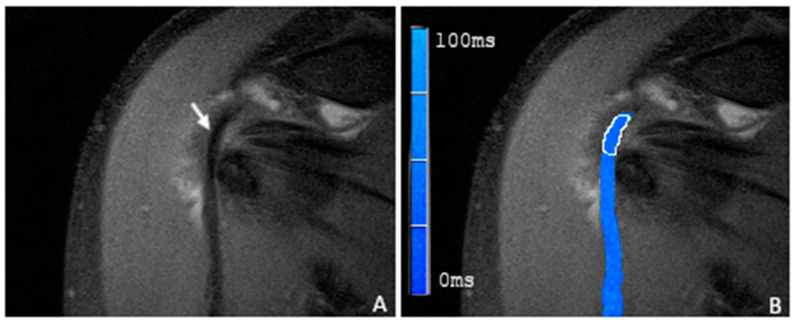
(**A**) A coronal proton-density-weighted fat-saturated magnetic resonance image of a 59-year-old man with morphologically normal-appearing and arthroscopically proven healthy tendon of the long biceps head (arrow). (**B**) A merged image of the proton-density-weighted images and the corresponding color-coded T2 maps with the placed ROI in the sulcal portion of the LBT (white frame). The average T2 mapping value was 21.3 ms.

**Figure 4:**
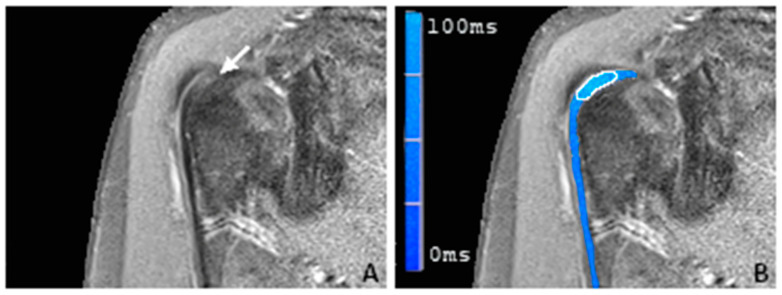
(**A**) A coronal proton-density-weighted fat-saturated magnetic resonance image of a 58-year-old man with focal hyperintensity of the sulcal portion of the LBT (arrow). (**B**) A merged image of the proton-density-weighted images and the corresponding color-coded T2 maps with the placed ROI in the sulcal portion of the LBT (white frame). Note the elevated average T2 mapping value of 44.7 ms. During the arthroscopy, a partial tear of the LBT was found.

**Figure 5:**
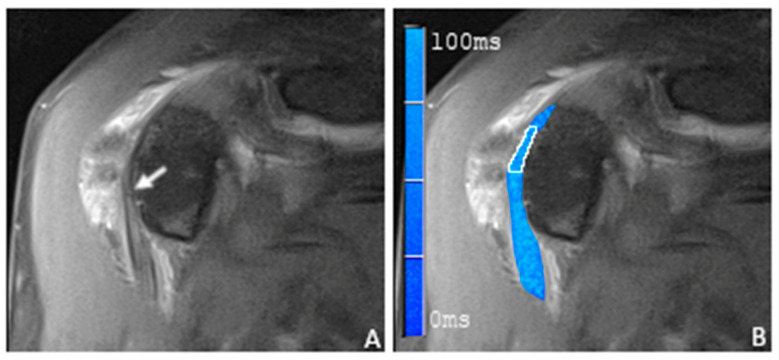
(**A**) A coronal proton-density-weighted fat-saturated magnetic resonance image of a 57-year-old man with longitudinal hyperintensity of the sulcal and the partially depicted extraarticular portion of the LBT with a markedly increased tendon diameter (arrow). (**B**) A merged image of the proton-density-weighted images and the corresponding color-coded T2 maps with the placed ROI in the sulcal portion of the LBT (white frame). Note the elevated average T2 mapping value of 52.8 ms. During the arthroscopy, a longitudinal partial tear was found.

In correlation with the statistical results, there was also a visual difference between the healthy subjects and the individuals with proven LBT pathologies in the color-coded T2 maps (Figure 3, Figure 4 and Figure 5).

### 3.3. Inter- and Intra-Reader Agreement

The calculation of the intra-class correlation coefficient (ICC) showed a near-perfect inter-reader agreement of 0.99 (95% confidence interval (CI): 0.98–1.00). The intra-reader agreement for the two respective readers was also almost perfect, with 0.93 (95% CI: 0.86–0.97) for reader 1 and 0.96 (95% CI: 0.97–0.99) for reader 2.

## 4. Discussion

Although pathologies of the long biceps tendon (LBT) are a well-known cause of shoulder pain, limited mobility, and associated impairments in daily living, common diagnostic tools have proven to be low in accuracy, particularly regarding the evaluation of early tendon damage. When detected by conventional MRI or MR arthrography, most LBT pathologies are visualized at more advanced stages of rupture or tendinopathy [7,8,9]. Despite the fact that tendon degeneration appears to have a chronically progressive course, diagnostic tools for early detection, although desirable, are not yet present [14]. T2 mapping as a functional and quantitative MRI sequence has been successfully used to evaluate the ultrastructural integrity of the 3D collagen network and water content of articular cartilage in several joints [16,17,29]. However, to our knowledge, its applicability to tendinous structures has not yet been systematically evaluated nor validated using arthroscopy as the gold standard.

Therefore, the aim of this study was to evaluate the ability of T2 mapping to identify and quantify pathologies of the LBT and to distinguish them from healthy tendons using arthroscopy as the gold standard. As one major finding, the mean T2 values showed a significant difference between the healthy subjects (23.3 ± 4.6 ms) and patients with LBT tendinopathy (47.9 ± 7.8 ms; *p* < 0.001). Additionally, the maximum T2 value in healthy subjects (29.6 ms) was lower than the minimum T2 value in damaged tendons (33.8 ms; *p* < 0.001). Therefore, these two findings, resulting in sensitivities, specificities, and positive and negative predictive values of 100% for all cut-off values between 29.6 and 33.8 ms (95% CI: 63.1–100.0%), demonstrate the ability of T2 mapping to evaluate tendinous tissues and to distinguish between healthy and damaged tendons. A previous study by our working group focused on the T2 mapping of SLAP lesions in the glenoid labrum [27]. The mean T2 values found in the pathological specimens differed to some extent from those reported in this study (37.7 ± 10.6 ms for SLAP lesions vs. 47.9 ± 7.8 ms for LBT pathologies), which could be explained by the different histological composition, being fibrocartilaginous for the glenoid labrum and mainly collagenous for the LBT. Nevertheless, as in this study, complete statistical separation of the T2 values was found between the healthy subjects and the patients with SLAP lesions. These significant differences in T2 relaxation time may reflect damages to the ultrastructural integrity of the collagen network and the associated biochemical repair mechanisms. In a histopathological correlation study, Buck et al. analyzed cadaveric tendon specimens from the LBT and compared the histological findings to the associated signal changes in MRI [23]. In addition to significant changes in the tendon diameter, the study group found mucoid degeneration with subsequent alterations in the water content as the main histopathological correlate for T2-signal hyperintensities in MRI. Another study showed that collagen network disorganization and myxoid/mucoid degeneration were the major histological correlates of tendon degeneration in 45 LBT specimens obtained from tenodesis [30]. These biochemical and histological processes are in line not only with the increased T2 relaxation times of the LBT shown in our study but also with several other studies describing an increase in the T2 values in damaged tendons other than the LBT or in the focal lesions of articular cartilage [31,32]. In a systematic review, Baum et al. described T2 mapping as a promising, non-invasive method for the biochemical evaluation of articular cartilage and fibrocartilaginous structures, like the menisci underlining the broad spectrum of potential clinical utility [33].

While T2 hyperintensities are well-known radiological correlates of tendon or cartilage damage using morphological MRI sequences, the novel aspect of T2 mapping lies in its ability to quantify the actual T2 relaxation times and thereby provide objectifiable measures. Thus, several areas of potential clinical use can be inferred. First, by providing cut-off values, quantification of the T2 relaxation times may help to increase diagnostic accuracy in the detection of tendon lesions. While the complete statistical separation between healthy and pathological tendon substance described in this study may be partially due to the small study population, future studies with larger sample sizes might show a gradual increase in T2 values with progressing degrees of degeneration and ascribe certain stages of degeneration to the associated levels of increased T2 relaxation times. In an analogy using area-under-the-curve calculations, Li et al. reported a diagnostic cut-off value of 49.5 ms for discriminating between healthy subjects and early stages of osteoarthritis in the articular cartilage of the knee (*n* = 79), with a resulting sensitivity of 91.2% and specificity of 92.3% [34]. However, larger sample sizes and additional studies of T2 mapping in tendinous tissue are needed to define reasonable cut-off values and thereby improve the diagnostic performance of MRI for detecting damage to the LBT.

Since there are currently no valid tools for the early detection of degenerative changes in collagen networks, T2 mapping, with its ability to detect and quantify ultrastructural damages and biochemical remodeling processes, holds promise for clinical use in the early detection of tendon pathologies. While explicit studies on early degenerative changes in the LBT are missing, several studies describe the general process of tendon degeneration as chronically progressive [15,35]. Analyzing the histopathological changes of 891 tendon specimens obtained from repair surgeries at various anatomic sites, Kannus et al. found that almost all (97%) tendons showed evidence of previous degeneration before tendon rupture occurred [36]. The study group also found similar degenerative changes in 34% of cadaveric tendon specimens from a young control group, indicating that tendon degeneration is a common finding in people older than 35 years. Although explicit studies on tendinous collagen networks are lacking, several studies have reported the ability of T2 mapping to sensitively detect early matrix degeneration in articular cartilage and predict its progression into manifest osteoarthritis (OA) [37,38]. Comparing normal controls and subjects with various risk factors for the development of OA but no radiographic signs of OA, Joseph et al. found higher and more heterogeneously distributed T2 values in subjects with OA risk factors than in the normal control group [39]. A similar but longitudinal study over a 24-month period reported higher T2 values in patients with risk factors for but without radiographic signs of OA compared to a normal control group [40]. As our study demonstrated the ability of T2 mapping to detect structural damage to the LBT, the aforementioned results may also be applicable to the T2 mapping of tendinous tissues. However, none of these studies used MRI for morphological correlation, and future studies need to further investigate the extent to which T2 mapping can detect early tendon degeneration, and thus, could serve as a valid tool for early detection in patients at risk for developing chronic tendinopathy. In particular, histological correlation studies using ex vivo human or animal models could be considered to further investigate the relationship between elevated T2 mapping values and histological changes. Additionally, diffusion-weighted sequences have also been described as a valid tool for the assessment of microstructural changes in musculoskeletal radiology. The combination of these sequences with quantitative T2 sequences might improve the detection of beginning histopathological changes even further than each of these techniques alone. The recently reported reduction in acquisition time using deep-learning-based reconstruction techniques in T2-weighted MR imaging may further enhance this advantage by allowing for the acquisition of additional information without negatively impacting the clinical workflow [41].

Another novel aspect yielded by the quantification of T2 relaxation times lies in its potential use for monitoring degenerative changes under conservative therapy or postoperative healing processes after tendon repair. Histological studies describe tendon healing as three overlapping phases consisting of an acute inflammatory phase with increased cellularity and vascularity and consequently increased water content, which, after a few days, transitions into the remodeling phase, in which the synthesis of glycosaminoglycans begins and the water content remains high. The last phase is described as the maturation/consolidation phase, during which the repair tissue changes from cellular to fibrous, and later, to scar-like tissue with a gradually decreasing water content [42]. Since T2 mapping evaluates the ultrastructural composition of collagen networks and changes in water content, it might provide objectifiable measures as correlates for these histological mechanisms. By evaluating the rotator cuff tendons after surgical repair over a 12-month period using T2 mapping, Xie et al. found gradually decreasing T2 values reaching the level of the healthy controls as a possible indicator of complete tendon healing [25]. The study group also described a correlation between lower T2 values and satisfactory clinical outcome after one-year follow-up. Another study focusing on the T2 values of knee cartilage after meniscal allograft transplantation reported similar results, with significantly increased T2 values in the early postoperative period that returned to baseline levels after one year [43]. Further studies correlating the longitudinal progression of T2 values with the clinical outcome after tendon, ligament, or cartilage repair could confirm these findings, thus qualifying T2 mapping as a method to further improve the clinical evaluation of postoperative healing processes by relating objectifiable measures to the common clinical examination.

In addition to the biochemical and histological factors discussed above, it is important to recognize that T2 mapping values may be influenced by several other variables that should be recognized as potential confounders. In addition to age-related changes in the composition of the tendon matrix and differences in mechanical loading, and consequently, water content along the entire tendon length, artificial signal changes, such as partial volume effects and the “magic angle” artifact, are known causes of variations in the T2 signal in various collagenous tissues [44,45]. The “magic angle” artifact is known to lead to a prolongation of T2 relaxation times in collagen fibers oriented at an angle of 55° to the magnetic field [46,47]. Because of its curved course through the glenohumeral joint, such imaging artifacts are particularly pronounced in the LBT. Buck et al. found signal changes caused by the “magic angle” artifact to be most eminent before the entrance of the LBT into the intertubercular groove [23]. They described the artificial signal intensity to be lower than T2 hyperintensities caused by histologically correlated mucoid degeneration. For this reason, and considering that the artifact of the “magic angle” can also lead to signal changes in healthy tendons, the significant differentiation in T2 values between the healthy subjects and the patients with confirmed tendinopathy observed in our study cannot be attributed to artificial factors only. The observed differentiation could possibly be due to the precise positioning of the region of interest (ROI) and the limited size of our study population. It is likely that future studies with larger cohorts will demonstrate overlap and a more gradual increase in T2 values, reflecting the progressive nature of LBT tendinopathy.

As evidenced by the almost perfect intra- and inter-reader agreement observed in the T2 values between the healthy and damaged long biceps tendons, T2 mapping demonstrates potential for reliable clinical utilization. The intraclass correlation coefficients (ICCs) for intra-reader agreement ranged from 0.93 to 0.96 (95% CI 0.86–0.99), while inter-reader agreement reached ICCs of up to 0.99 (95% CI 0.98–1.00). In concordance with this, previous studies on T2 mapping of the glenohumeral joint in healthy subjects have shown comparable intraclass correlation coefficients (ICCs), with good to excellent agreement among investigators [48,49]. Nevertheless, it is important to acknowledge that the small sample size and the limited number of confirmed LBT lesions (*n* = 8) in our study could potentially contribute to statistical artifacts, which may have influenced these findings to some extent.

Our study has some limitations. As a major limitation, the small sample size has to be mentioned, as it has important statistical implications. In studies investigating a small number of participants, extreme values can have a disproportionate impact on confidence interval (CI) calculations, resulting in wider ranges. For this study, the 95% CI for sensitivity and specificity ranged from 63.1% to 100%, indicating the potential effect of the small sample size on the precision of these measures. Furthermore, this study recruited patients from a specialized referral center, which makes it highly selective and prone to sampling errors. The higher prevalence of LBT lesions in this specific population may have led to improved test performance. Given these statistical limitations, the results may apply only to our specific study cohort, giving this study the characteristics of a pilot or preliminary study. To address this concern, further studies with larger sample sizes are necessary. These larger studies would also provide the opportunity to more accurately differentiate lesion severity and potentially identify different T2 values for the respective lesion grades.

Another limitation to consider is the possibility of human error in the manual placement of regions of interest (ROIs), which can lead to variability and subjective judgments. Although all patients were positioned for MRI examination in the same way and a standardized slice orientation was used for ROI placement (Figure 1), the exact position of ROI placement differed slightly among the subjects because of small interindividual variations in anatomy. Despite our efforts to mitigate potential errors arising from the imprecise placement of the region of interest (ROI) by averaging T2 values obtained from three different measurements, there is still room for improvement. Additionally, it has to be mentioned that the long acquisition time of the T2 mapping sequences (Table 1) is susceptible to motion artifacts, which in turn, could influence T2 mapping values. In future studies, the utilization of 3D T2 mapping sequences with voxel-wise analysis of T2 relaxation times would be highly advantageous. This advanced approach would enable the most precise acquisition of T2 values, enhancing the accuracy and reliability of the results. In addition, 3D sequences could also reduce the potential impact of the partial volume effect in the imaging of the LBT, which could be better visualized than with conventional 2D sequences due to its arcuate course combined with its usually small size. Examination of the LBT using dynamic modalities, such as ultrasound, could also contribute to a better assessment of tendon substance and reveal correlations between sonographic findings and elevated T2 values. Furthermore, because the intertubercular portion of the LBT may be partially invisible to arthroscopy, it is possible that small tendon portions captured within the ROI may have missed arthroscopic correlation. However, in correspondence with orthopedic surgeons and careful review of the surgical protocols, all lesions found during the arthroscopy were longitudinally distributed along the course of the tendon, and thus, were reliably detectable.

Additionally, it is important to note that, to the best of our knowledge, this study represents the first systematic evaluation of T2 mapping specifically for the LBT. As a result, there are no suitable studies available for direct comparison of the quantified T2 relaxation times in this context. The few existing studies on the T2 mapping of tendinous structures have reported similar results, with T2 relaxation times around 30 ms for healthy supraspinatus tendons, for instance [24,25]. However, further research is required to validate the T2 mapping values specifically for the long biceps tendon, as observed in our study.

Finally, it should be mentioned that the T2 values analyzed in this study and the resulting diagnostic parameters are representative only of a field strength of 3.0 Tesla. Some studies comparing cardiac and uterine T2 mapping sequences at 1.5 and 3.0 Tesla found significant field strength-dependent differences in the mean T2 values [50,51]. While explicit studies on the cartilaginous tissue of tendinous structures are lacking, this effect might also be applicable to musculoskeletal imaging.

## 5. Conclusions

T2 mapping effectively detects and quantifies long biceps tendon (LBT) pathologies, as evidenced by the significantly higher T2 values observed in arthroscopically confirmed tendinopathy compared to provenly healthy tendons. Additionally, the high diagnostic performance values and strong inter-rater reliability (ICC) among radiologists further support this finding. Thus, T2 mapping has the potential to provide valuable insights into the (ultra-)structural integrity of the tendinous collagen network and may, therefore, serve as a valuable tool for early diagnosis, facilitating the prompt initiation of therapy. Additionally, by providing objectifiable measures, it may enable longitudinal quantitative monitoring during conservative therapy or after surgical treatment.

## Figures and Tables

**Table 1:** In-house shoulder MRI protocol and T2 mapping study sequence.

No.	Sequence	Orientation	Repetition Time (TR; ms)	EchoTime (TE; ms)	Acquisition Matrix	Flip Angle	Echo Train Length	No. of Slices	TA (min)	Slices (mm)
1	PD FS TSE	axial	3660	24	384 × 346	176	7	27	04:32	3
2	PD FS TSE	oblique coronal	2490	24	384 × 307	160	7	19	03:37	3
3	PD FS TSE	oblique sagittal	3950	23	320 × 256	140	7	29	04:49	3
4	PD TSE	oblique coronal	1670	23	384 × 307	160	5	19	03:24	3
5	T1 SE	oblique coronal	787	10	384 × 346	90	1	19	04:51	3
6	T2 TSE	oblique sagittal	5640	88	384 × 307	150	15	29	02:33	3
7	T2 MapIt	oblique coronal	2140	13.8, 27.6, 41.4, 55.2, 69	320 × 320	180	1	16	06:50	3

TA = time of acquisition, PD = proton-density, FS = fat-saturated, TSE = turbo spin-echo. Note: Differences in the values of the acquisition matrix and flip angle were caused by differences in the imaged volume among the respective slice orientations.

**Table 2:** Demographic characteristics for patients with and without LBT lesion.

		LBS Tendinopathy		
		No (N = 10)	Yes (N = 8)	*p*-Value
Sex	Male	5 (50.0%)	7 (87.5%)	
	Female	5 (50.0%)	1 (12.5%)	
Age (years)	*n*	10	8	0.1274
	Mean	46.3	60.0	
	SD	17.24	5.01	
	Median	54.0	59.5	
	Min	22.0	52.0	
	Max	64.0	67.0	

SD = standard deviation, Min = minimum, Max = maximum.

**Table 3:** T2 mapping values (ms) for normal and damaged LBT.

		Overall	Population without Lesion	Population with Lesion	*p*-Value (t Value)
	n	18	10	8	
T2 values (ms)	Mean	34.2	23.3	47.9	<0.001 (−8.33)
	SD	13.97	4.61	7.84	
	Median	29.0	24.2	48.2	
	Min	15.8	15.8	33.8	
	Max	61.4	29.6	61.4	

SD = standard deviation, Min = minimum, Max = maximum.

## Data Availability

The datasets generated and analyzed in the present study are available from the corresponding author upon reasonable request.
